# Age-independent rise of inflammatory scores may contribute to accelerated aging in multi-morbidity

**DOI:** 10.18632/oncotarget.2725

**Published:** 2015-02-10

**Authors:** Maria Stepanova, Edgar Rodriguez, Aybike Birerdinc, Ancha Baranova

**Affiliations:** ^1^ Center for the Study of Chronic Metabolic Diseases, School of System Biology, George Mason University, Fairfax, VA, USA; ^2^ Betty and Guy Beatty Center for Integrated Research, Inova Health Systems, Falls Church, VA, USA; ^3^ Research Center for Medical Genetics RAMS, Moscow, Russian Federation; ^4^ Atlas Biomed Group, Moscow, Russia

**Keywords:** multi-morbidity, C-reactive protein, systemic inflammation, Glasgow Prognostic Score, aging

## Abstract

Aging is associated with an increase in a chronic, low-grade inflammation. This phenomenon, termed “inflammaging” is also a risk factor for both morbidity and mortality in the elderly. Frequent co-occurrence of chronic diseases, known as multi-morbidity, may be explained by interconnected pathophysiology of these conditions, most of which depend on its inflammatory component. Here we present an analysis of the U.S. National Health and Nutrition Examination Survey data collected between 1999 and 2008, for the presence, and the number, of chronic diseases along with HDL-cholesterol, C-reactive protein, white blood cell count, lymphocyte percent, monocyte percent, segmented neutrophils percent, eosinophils percent, basophils percent, and glycohemoglobin levels. Importantly, even after adjustment for age and BMI, many inflammatory markers continued to be associated to multi-morbidity. C-reactive protein (CRP) levels and Glasgow Prognostic Score (GPS) were most dramatically increased in parallel with an accumulation of chronic diseases, and may be utilized as multi-morbidity predictors. These observations point at background inflammation as direct, age-independent contributor to an accumulation of the disease burden. Our findings also suggest a possibility that systemic inflammation associated with chronic diseases may explain accelerated aging phenomenon previously observed among the patients with heavy disease burden.

## INTRODUCTION

Multi-morbidity, which is defined as the coexistence of multiple chronic diseases or conditions in an individual, is a prevalent clinical reality especially found in adult and elderly patients. In some cases, multi-morbidity may be recognized as a clinical entity. An example of a multi-morbid condition, metabolic syndrome (MS) is diagnosed when central obesity and any two of four other factors (elevated triglycerides, reduced high density lipoprotein cholesterol, elevated blood pressure, elevated fasting plasma glucose) are observed [1]. In turn, this condition conveys an increased risk of other diseases such as cardiovascular disease (CVD) [2]. Both MS and CVD are known contributors to aging. In fact, these pathological conditions are associated with the same physiological changes as these observed in aging, including an increase in intensity of free radical generating intracellular processes [3] and in chronic low-grade inflammation, known as Inflamm-Aging [4].

Another common multi-morbid condition is obesity that is known for its association with both MS and CVD, but also for less a number of chronic diseases with less obvious links to excessive adiposity, including arthritis, asthma and cancer [5, 6]. Recent study showed that obesity was associated with more than double the odds of multi-morbidity as compared to non-obese cohort [7]. Frequent co-occurrence of chronic diseases may be explained by both cause-consequence relationships and interconnected pathophysiology. In particular, low-grade chronic inflammation, a hallmark of central obesity, is a common finding in patients with cardiovascular disease, diabetes, hypertension, non-alcoholic fatty liver disease, and some cancer [8]. In obese individuals, the vicious cycle of inflammation is propagated by the innate immune system activation within adipose, which, in turn, promotes the production and release of pro-inflammatory cytokines that fuel the systemic acute phase-responses [9].

On the other hand, a low-grade inflammatory condition may also develop in non-obese individuals. For example, hyperandrogenism related to polycystic ovary syndrome (PCOS) is a known stimulator of the NF-kB inflammatory pathway in lean women of reproductive age [10]. Oxidative stress due to either increased internal production of reactive oxygen species (ROS) or environmental exposure was causatively linked to both elevated inflammatory background in the human body [11] and an activity of autoimmune diseases [12]. Another factor that contributes to systemic inflammation includes the production of endogenous lipopolysaccharide (LPS) by the gut microbiota and resultant “metabolic endotoxemia” that, in turn, contributes to insulin resistance, and augments cardiovascular risk [13, 14].

In this study, we employed National Health and Nutrition Examination Survey (NHANES) population datasets in an attempt to determine an association of inflammation-related parameters with the prevalence of individual chronic diseases and various types of multi-morbidity. Our study demonstrates that even after adjustment for age and BMI, many inflammatory markers, including C-reactive protein and Glasgow score, continued to be associated to multi-morbidity, and increase in parallel with an accumulation of chronic diseases. The results of our study contribute some explanation to previously reported findings of accelerated biological aging in patients with chronic disease burden.

## RESULTS

### Study population

Of all 51,623 NHANES participants enrolled by the Centers for Disease Control (CDC) between 1999 and 2008, only 26,225 (weighted population count 208,503,656) were considered eligible for the study. Of those, 7,123 (28.07 ± 0.37%) individuals had only one chronic disease, 9,992 (36.74 ± 0.69%) individuals had two or more chronic diseases, and 9,110 (35.19 ± 0.68%) individuals were considered healthy controls. The average number of chronic diseases in the multi-morbidity group was 3.017 ± 0.021.

### Demographic markers

The most relevant demographic differences between individuals with and without chronic diseases are summarized in Table 1. Individuals with chronic diseases were relatively older. Of the individuals with only one chronic disease, approximately one-half were age 45 and younger (57.34 ± 1.02%) while of those with multi-morbidity, approximately one-quarter of the individuals with two or more chronic diseases were age 45 and younger (28.25 ± 0.84). The average age of those without chronic disease was 36.85 ± 0.26 years, of those with one chronic disease was 42.76 ± 0.30 years, and of those with two or more chronic diseases was 54.50 ± 0.31 years (Table 1).

**Table 1:** Demographic and clinical parameters of individuals without chronic diseases, with one chronic disease, and with multi-morbidity

Sample size and Parameter	Healthy controls	One chronic disease	Multi-morbidity	*p*
Sample number	9,110	7,123	9,992	NA
Population prevalence, %	35.19 +/− 0.68	28.07 +/− 0.37	36.74 +/− 0.69	NA
Age, years	36.850 +/− 0.241	42.764 +/− 0.296	54.500 +/− 0.319	< 0.0001
Caucasian, %	66.71 +/− 1.85	69.60 +/− 1.97	75.45 +/− 1.67	< 0.0001
African-American, %	10.22 +/− 0.89	11.79 +/− 1.06	12.22 +/− 1.16	0.0005
Hispanic, %	16.71 +/− 1.35	14.12 +/− 1.38	8.80 +/− 0.98	< 0.0001
Other ethnicity, %	6.36 +/− 0.54	4.49 +/− 0.42	3.53 +/− 0.38	< 0.0001
Male, %	53.67 +/− 0.57	49.46 +/− 0.66	42.46 +/− 0.57	< 0.0001
College degree, %	29.16 +/− 1.23	25.48 +/− 1.08	20.33 +/− 0.87	< 0.0001
Married, %	51.55 +/− 0.98	57.39 +/− 1.12	60.43 +/− 0.91	< 0.0001
Hypercholesterolemia, %	24.46 +/− 0.89	34.32 +/− 0.84	51.73 +/− 0.76	< 0.0001
Alcohol consumption, %	10.94 +/− 0.54	8.97 +/− 0.54	7.59 +/− 0.42	< 0.0001
Smoking, %	32.59 +/− 1.16	33.14 +/− 1.08	32.46 +/− 0.95	0.82
Injection drug use history, %	2.99 +/− 0.42	2.99 +/− 0.40	2.71 +/− 0.26	0.71

Other demographic parameters described in Table 1 that exhibit significant differences between those with and without chronic diseases could, at least partially, be associated with this age disparity. Caucasian individuals accounted for 66.71 ± 1.85% of the healthy control population, accounted for 69.60 ± 1.97% of individuals with one chronic disease, and accounted for 75.45 ± 1.67% of individuals with two or more chronic diseases. In contrast, as described in Table 1, Hispanic individuals accounted for 16.71 ± 1.85% of the healthy control population, accounted for 14.12 ± 1.38% of individuals with one chronic disease, and accounted for 8.80 ± 0.98% of individuals with two or more chronic diseases.

### Co-occurrence of the chronic diseases and multi-morbidity trends

Of all the individually studied chronic diseases (Table 2), the most prevalent in the total U.S. population were obesity (31.87 ± 0.61%), hypertension (26.93 ± 0.57%), arthritis (22.98 ± 0.61%) and asthma (13.09 ± 0.34%), followed by thyroid disease (8.91 ± 0.27%) and history of cancer (7.98 ± 0.29%). The least prevalent were kidney failure (2.13 ± 0.12%) and CHF (2.17 ± 0.11%). Since hypercholesterolemia is known to be a reversible, asymptomatic condition, the hypercholesterolemic individuals remained in the control population for the purpose of current analysis. However, the percentages of the patients with hypercholesterolemia increased along with accumulation of chronic diseases; this condition was diagnosed in 24.46 ± 0.89% of the healthy control population, 34.32 ± 0.84% of individuals with one chronic disease and 51.73 ± 0.76% of individuals with two or more chronic diseases.

**Table 2:** Clinical diagnoses in individuals with one chronic disease and with multi-morbidity

Diagnosis	One chronic disease	Multi-morbidity	*p*
Asthma, %	13.93 +/− 0.59	24.99 +/− 0.69	< 0.0001
Arthritis, %	13.79 +/− 0.60	50.29 +/− 0.82	< 0.0001
Cancer, %	4.35 +/− 0.31	17.81 +/− 0.61	< 0.0001
Congestive heart failure %	0.13 +/− 0.05	5.62 +/− 0.26	< 0.0001
Chronic liver disease	2.28 +/− 0.25	7.00 +/− 0.34	< 0.0001
^a^COPD, %	3.00 +/− 0.31	18.50 +/− 0.64	< 0.0001
Diabetes, %	2.43 +/− 0.27	17.87 +/− 0.56	< 0.0001
Hypertension, %	17.04 +/− 0.55	60.03 +/− 0.72	< 0.0001
Ischemic heart disease, %	17.04 +/− 0.55	60.03 +/− 0.72	< 0.0001
Kidney failure, %	17.04 +/− 0.55	60.03 +/− 0.72	< 0.0001
Obesity, %	37.30 +/− 0.89	58.15 +/− 0.76	< 0.0001
Stroke, %	0.29 +/− 0.07	6.39 +/− 0.29	< 0.0001
Thyroid disease, %	5.53 +/− 0.39	19.35 +/− 0.59	< 0.0001
Number of chronic diseases	1	3.017 +/− 0.021	NA

a Chronic obstructive pulmonary disease

Strikingly, all studied chronic diseases exhibited a dramatic trend for co-occurrence. For example, the prevalence of obesity was at 37.30 ± 0.89% in individuals with one chronic disease and 58.15 ± 0.76% in individuals with two or more chronic diseases.

### Inflammatory markers

All of the assessed direct or indirect biomarkers of inflammation, including HDL-cholesterol, C-reactive protein, white blood cell count, lymphocyte percent, monocyte percent, segmented neutrophils percent, eosinophils percent, basophils percent, glycohemoglobin, and Glasgow Prognostic Scores (GPS) were statistically different when healthy controls were compared to those with one chronic disease or with two or more chronic diseases (Table 3). Lymphocyte percent, monocyte percent and HDL decreased with a progression to multi-morbidity, while all other biomarkers increased. This increase was especially striking for C-reactive protein levels that were at 0.265 ± 0.011 mg/dl, at 0.392 ± 0.011 mg/dl and at 0.580 ± 0.012 mg/dl in a cohort without chronic diseases, a cohort with one disease, and a cohort with multi-morbidity, respectively. In a cohort with one disease and in multi-morbid cohort, the percentages of individuals with GPS scores of 1 or 2 were significantly larger than that non-diseased cohort (Table 3).

**Table 3:** Inflammatory markers in individuals without chronic disease, with one chronic disease, and with two or more chronic diseases

Clinical parameter	Healthy controls	One chronic disease	Two or more chronic diseases	*p*
^a^HOMA	2.066 +/− 0.043	3.064 +/− 0.085	4.762 +/− 0.108	< 0.0001
^b^HDL-cholesterol, mg/dL	54.569 +/− 0.315	51.772 +/− 0.304	51.112 +/− 0.301	< 0.0001
^c^CRP, mg/dL	0.265 +/− 0.011	0.392 +/− 0.011	0.580 +/− 0.012	< 0.0001
^d^WBC count, SI	7.070 +/− 0.039	7.366 +/− 0.040	7.509 +/− 0.036	< 0.0001
Lymphocyte, %	30.328 +/− 0.131	30.054 +/− 0.149	29.381 +/− 0.131	< 0.0001
Monocyte, %	7.963 +/− 0.037	7.864 +/− 0.043	7.872 +/− 0.034	0.0155
^e^Sn, %	58.367 +/− 0.149	58.659 +/− 0.172	59.134 +/− 0.136	0.0015
Eosinophils, %	2.719 +/− 0.029	2.796 +/− 0.030	2.957 +/− 0.023	< 0.0001
Basophils, %	0.657 +/− 0.009	0.663 +/− 0.010	0.691 +/− 0.008	0.0054
Glycohemoglobin, %	5.189 +/− 0.010	5.390 +/− 0.015	5.796 +/− 0.016	< 0.0001
^f^GPS=0, % in cohort	94.13 +/− 0.35	90.51 +/− 0.43	82.93 +/− 0.52	< 0.0001
GPS=1, % in cohort	5.58 +/− 0.34	8.91 +/− 0.42	16.04 +/− 0.53	< 0.0001
GPS=3, % in cohort	0.29 +/− 0.06	0.58 +/− 0.09	1.03 +/− 0.11	< 0.0001

a Homeostatic model assessment;

b High density lipoprotein;

c C-reactive protein;

d White blood cell count;

e Segmented neutrophils;

f Glasgow Prognostic Scores

After an adjustment for age and BMI, only HOMA score, C-reactive protein, white blood cell count, lymphocyte percent, segmented neutrophils percent, eosinophils percent and glycohemoglobin levels were found to be associated to multi-morbidity expressed as chronic disease number (Table 4). Of these, HOMA score, C-reactive protein, white blood cell count, segmented neutrophils percent, eosinophils percent and glycohemoglobin levels were found to be positively associated with chronic disease number while lymphocyte percent was found to be negatively associated with chronic disease number.

**Table 4:** Association of inflammatory markers with any chronic disease and with an accumulation of chronic diseases after adjustment for age and body mass index

Clinical parameter	Number of chronic diseases (95% CI)	Any chronic disease, (95% CI)
^a^HOMA	0.0390 (0.0312 – 0.0468) @	1.0634 (1.0423 – 1.0850) @
^b^HDL-cholesterol (mg/dL)	−0.0004 (–0.0016 – 0.0009)	1.0018 (0.9991 – 1.0044)
C-reactive protein (mg/dL)	0.1040 (0.0741 – 0.1339) @	1.1389 (1.0658 – 1.2170) @
White blood cell count (SI)	0.0293 (0.0209 – 0.0376) @	1.0452 (1.0260 – 1.0647) @
Lymphocyte (%)	−0.0055 (–0.0077 – 0.0033) @	0.9943 (0.9900 – 0.9987)
Monocyte (%)	−0.0066 (–0.0142 – 0.0010)	0.9855 (0.9693 – 1.0020)
Segmented neutrophils (%)	0.0033 (0.0014 – 0.0052) @	1.0028 (0.9990 – 1.0067)
Eosinophils (%)	0.0265 (0.0171 – 0.0360) @	1.0448 (1.0273 – 1.0627) @
Basophils (%)	0.0198 (–0.0180 – 0.0576)	1.0883 (1.0081 – 1.1750)
Glycohemoglobin (%)	0.2340 (0.2024 – 0.2656) @	1.3836 (1.3087 – 1.4627) @

a Homeostatic model assessment

b High density lipoprotein

@ – *p*-value < 0.05 after multiple test correction

### C-reactive protein levels in patients with cardiovascular and non-cardiovascular morbidities

Remarkably, the levels of CRP in patients with any cardiovascular disease defined as hypertension, ischemic heart conditions, coronary heart disease or stroke were not substantially different from that in non-cardiovascular diseases. Specifically, when cohort with only one condition was stratified into cardiovascular disease and non-cardiovascular morbidity cohorts, the levels of CRP were similar in those with and without cardiovascular disease: 0.369 ± 0.029 mg/dL vs. 0.399 ± 0.011 mg/dL (*p* = 0.33). In similarly stratified multi-morbidity cohort, observed differences in CRP levels were 0.592 ± 0.016 mg/dL vs. 0.557 ± 0.017 mg/dL (*p* = 0.14). These findings are in contrast with striking increases in C-reactive protein levels observed along with accumulation of overall disease burden and described above.

## DISCUSSION

Our analysis of NHANES cohorts detected multi-morbidity in 36.74% of adult Americans, which indeed makes multi-morbidity the most prevalent chronic condition in the U.S. population. The co-occurrence of chronic diseases is of dramatic proportions. At least in part these trends could be explained by a known phenomenon of disease accumulation with age and are in agreement with previous reports [15, 16]. Although more than one-half of the individuals with one chronic disease were age 45 and younger (57%), the average age for the group was 42.8 years placing the typical onset of single morbidity in the fifth decade of life. The average age for individuals with two or more chronic diseases was 54.5 ± 0.32 years (Table 1). Thus, higher age was a strong demographic predictor of chronic disease number and their prevalence increased with age.

However, even after adjustment for age and BMI, a number of inflammation-related parameters, including Glasgow Prognostic Score (GPS) [17], retained its high significance within the model that describe an accumulation of morbid conditions in U.S. population, thus, pointing at a key role of systemic inflammation in the development of multi-morbidity. In this model, the percent of glycohemoglobin and the levels of C-reactive protein were the strongest contributors to morbidity loads (Table 4).

C-reactive protein (CRP) participates in the systemic response to inflammation and its levels are known to be proportional to cardiovascular risks [18]. This marker also had been extensively used to predict mortality in non-cardiovascular conditions, for example, in COPD [19], polycystic ovary disease (PCOS) [20], chronic kidney disease [21] and others. In our study, observed cross-cohort increase in the levels of C-reactive protein was especially striking when contrasted to the lack of difference in levels of CRP when patients with any cardiovascular disease were compared to the cohort with any non-cardiovascular morbidity. This observation indicates that an association of CRP levels with a chronic disease burden seems to be a component of an overall increase in systemic inflammation rather than a reflection of an overall decrease in the health of heart and blood vessels in multi-morbid populations.

Another inflammation-related prognostic measure, The Glasgow Prognostic Score (GPS), ties together the levels of C-reactive protein and albumin [17]. In a number of studies, GPS scores were shown to be instrumental in making informed decisions on whether to proceed with antitumor chemotherapy in frail patients [22], in predicting clinical outcomes of hemodyalisis [23] and in both resectable and metastatic cancers [reviewed in 24]. In our study, the inflammatory GPS scores were not useful as predictors of multimorbidity, as only small percentages of each cohort had GPS above zero. However, the observed percentages of multi-morbid individuals with GPS scores of 1 (16.04 +/− 053) and 2 (1.03 +/− 0.11) are disturbing, as these scores are directly associated with frailty-associated mortality. Hence, our study points at possible extension of GPS score applicability in assessment of multi-morbid individuals with non-malignant disorders.

Additionally, patients with multi-morbidities had higher homeostatic model assessment (HOMA) scores that determine the degree of insulin resistance, and the levels of glucosylated hemoglobin that reflect glycemic control. Both measures were earlier shown to correlate to levels of CRP; moreover, an increase in CRP levels is a known risk factor for type 2 diabetes [25, 26]. However, later studies that employed Mendelian randomization approach showed that observed associations between serum CRP and insulin resistance, glycemia, and diabetes are likely to be non-causal [27] and suggest that both the levels of CRP and HOMA scores are being propped by upstream inflammatory reactions. This conclusion is in agreement with the results of current study that suggests a key role of the systemic inflammation in accumulation of subsequent morbidities.

Given that morbid burden increases with age, and that aging itself is associated with increased levels of proinflammatory markers due to immunosenescence [28], one may ask whether an increase in inflammation is a primary contributor to the development of multi-morbidity. To address this possibility, we performed an adjustment of prevalence data for both age and BMI, factors known to both increase with age and contribute to systemic inflammation. After this adjustment, HOMA score, C-reactive protein, white blood cell count, lymphocyte percent, segmented neutrophils percent, eosinophils percent and glycohemoglobin levels continued to be associated to multi-morbidity, thus confirming our hypothesis that background inflammation directly promotes an accumulation of the disease burden in an age-independent manner. As a corollary to this hypothesis, we suggest a possibility that systemic inflammation associated with chronic diseases may directly contribute to accelerated aging observed among the patients with heavy disease burden.

## METHODS

This study analyzed five cycles of National Health and Nutrition Examination Survey (NHANES) data collected between 1999 and 2008. Each NHANES cycle includes data on approximately 10 thousand unique non-institutionalized United States residents with no participant being included twice in any two cycles. In this study, only NHANES participants of 18 years of age or older were included.

For eligible participants, the demographic parameters of age, gender, marital status, education level, and ethnicity were collected from NHANES Demographics questionnaires. Ethnicity was encoded as Caucasian, African-American, Hispanic, or Other, which included Aleut, Eskimo, American Indian, Asian, and Pacific Islander.

The clinical parameters used in this study were reported as follows. Obesity was defined as body mass index (BMI) of 30 or higher, hypercholesterolemia was defined as an elevated cholesterol of greater than 200 mg/dL, LDL greater than 139 mg/dL or HDL less than 40 mg/dL for men and less than 50 mg/dL for women [29]. The HOMA scores were calculated using the homeostasis assessment model, fasting glucose and insulin levels [30]. The list of assessed biomarkers related to inflammation included HDL-cholesterol, C-reactive protein, white blood cell count, lymphocyte percent, monocyte percent, segmented neutrophils percent, eosinophils percent, basophils percent, and glycohemoglobin. The Glasgow Predictive Scores (GPS) were calculated according to [17]. Briefly, patients with both an elevated C-reactive protein (> 10 mg/L) and hypoalbuminaemia (< 35 g/L) were allocated a score of 2. Patients in whom only one of these biochemical abnormalities was present were allocated a score of 1. Patients in whom neither of these abnormalities was present were allocated a score of 0.

### Chronic diseases

The presence, and the number, of chronic diseases were determined for all NHANES participants who completed the Medical Conditions questionnaires. The list of studied chronic diseases included history of: *asthma*; *arthritis* (any type); *cancer* (any type); *congestive heart failure (CHF)*; *chronic liver disease* (*CLD*, any type); *chronic obstructive pulmonary disease* (*COPD*, which included history of chronic bronchitis or emphysema); *ischemic heart disease* (*IHD*, which included history of coronary artery disease, angina or heart attack); *stroke*; *thyroid disease* (any type); history of *type II diabetes* (*DM*, collected from Diabetes questionnaires); *hypertension* (Blood Pressure and Cholesterol questionnaires); and *kidney failure* (Urology and Kidney Conditions questionnaire). Obesity was excluded from the list of chronic diseases studied for chronic disease number after adjustment for BMI. However, the association of the studied inflammatory factors with obesity alone was examined. Thus, for a total of 12 chronic diseases forming a multi-morbidity contributors list, the association of demographic and clinical parameters with chronic disease number was studied after adjustment for BMI. An individual association of each of these diseases and of obesity with demographical and clinical characteristics was also assessed. Individuals with no chronic diseases were used as controls.

### Statistical analysis

As recommended by the NHANES Analytic and Reporting Guidelines [31], to make the distribution of the participants representative of that of the U.S. population, sampling weights were used to account for non-response and unequal selection probabilities for certain categories of the population. Additionally, stratum and sampling units accounted for the survey design effects using Taylor series linearization. Furthermore, when merging NHANES study cycles, appropriate selection of sampling weights and adjustment coefficients were applied. Finally, where ascertained due to changes in laboratory techniques, the recommended adjustment of laboratory parameters was made to make the data comparable between the study cycles.

Pairwise comparisons between chronic disease groups and healthy controls were performed using the stratum-specific chi-square test for independence (for binary parameters such as gender) and a t-test for a contrasted mean (for continuous parameters such HOMA score). Similarly, individuals with a chronic disease were compared to healthy controls for each studied chronic disease separately. To calculate the association of inflammatory markers and chronic diseases (any and each separately) adjusted for age and BMI, logistic regression was used (linear regression for the outcome being the number of chronic diseases). Unless stated otherwise, *p*-values of 0.05 or less after Benjamini-Hochberg multiple test correction were considered potentially statistically significant. All statistical analyses were run using SUDAAN 10.0 (SAS Institute Inc., Cary, NC).
